# Health Care Provider Counseling for Weight Loss Among Adults with Arthritis and Overweight or Obesity — United States, 2002–2014

**DOI:** 10.15585/mmwr.mm6717a2

**Published:** 2018-05-04

**Authors:** Dana Guglielmo, Jennifer M. Hootman, Louise B. Murphy, Michael A. Boring, Kristina A. Theis, Brook Belay, Kamil E. Barbour, Miriam G. Cisternas, Charles G. Helmick

**Affiliations:** ^1^Division of Population Health, National Center for Chronic Disease Prevention and Health Promotion, CDC; ^2^Oak Ridge Institute for Science and Education (ORISE); ^3^Division of Nutrition, Physical Activity and Obesity, National Center for Chronic Disease Prevention and Health Promotion, CDC.

In the United States, 54.4 million adults report having doctor-diagnosed arthritis (*1*). Among adults with arthritis, 32.7% and 38.1% also have overweight and obesity, respectively (*1*), with obesity being more prevalent among persons with arthritis than among those who do not have arthritis (*2*). Furthermore, severe joint pain among adults with arthritis in 2014 was reported by 23.5% of adults with overweight and 31.7% of adults with obesity (*3*). The American College of Rheumatology recommends weight loss for adults with hip or knee osteoarthritis and overweight or obesity,* which can improve function and mobility while reducing pain and disability (*4*,*5*). The *Healthy People 2020* target for health care provider (hereafter provider) counseling for weight loss among persons with arthritis and overweight or obesity is 45.3%.^†^ Adults with overweight or obesity who receive weight-loss counseling from a provider are approximately four times more likely to attempt to lose weight than are those who do not receive counseling (*6*). To estimate changes in the prevalence of provider counseling for weight loss reported by adults with arthritis and overweight or obesity, CDC analyzed National Health Interview Survey (NHIS) data.^§^ Overall, age-standardized estimates of provider counseling for weight loss increased by 10.4 percentage points from 2002 (35.1%; 95% confidence interval [CI] = 33.0–37.3) to 2014 (45.5%; 95% CI = 42.9–48.1) (p<0.001). Providing comprehensive behavioral counseling (including nutrition, physical activity, and self-management education) and encouraging evidence-based weight-loss program participation can result in enhanced health benefits for this population.

NHIS is an ongoing, in-person, cross-sectional survey of the civilian, noninstitutionalized population. CDC analyzed data on adults aged ≥18 years with arthritis and overweight or obesity from the Sample Adult component for 2002, 2003, 2006, 2009, and 2014 (24,275–36,697; response rate = 58.9%–74.3%). Having arthritis was defined as an affirmative response to the question “Have you ever been told by a doctor or other health care professional that you have arthritis, rheumatoid arthritis, gout, lupus, or fibromyalgia?” Body mass index (BMI), defined as weight (kg) divided by height (m^2^), was calculated from self-reported height and weight and categorized as: normal/underweight (<25); overweight (25 to <30); and obese (≥30).^¶^ Obesity was further stratified into three BMI subgroups: class 1 (30 to <35); class 2 (35 to <40); and class 3 (≥40).** Provider counseling for weight loss, which was part of sponsored survey content featured in 2002, 2003, 2006, 2009, and 2014, was defined as an affirmative response to the question, “Has a doctor or other health professional ever suggested losing weight to help your arthritis or joint symptoms?”

All analyses accounted for the complex survey design; sampling weights were applied to make estimates representative of the U.S. civilian, noninstitutionalized population. Weighted numbers and age-standardized prevalences (using the projected 2000 U.S. population for ages 18–44, 45–64, and ≥65 years)^††^ were calculated for adults with overweight or obesity overall and for selected sociodemographic and health-related characteristics for 2002 and 2014. Results were declared significant if t-tests yielded p-values <0.05 for differences in age-standardized prevalences between 2002 and 2014, and between categories of characteristics in 2014.

Among the U.S. adult population, 28.3 million persons in 2002 and 38.9 million in 2014 had arthritis and overweight or obesity. From 2002 to 2014, the age-standardized prevalence of receiving provider counseling for weight loss among adults with arthritis and overweight or obesity increased by 10.4 percentage points from 35.1% (95% CI = 33.0–37.3) to 45.5% (95% CI = 42.9–48.1) (p<0.001) (Table), which met the *Healthy People 2020* target of 45.3%. The prevalence increased by 5.7 percentage points for adults with arthritis and overweight (from 18.1% to 23.8%; p = 0.006) and 12.4 percentage points for those with obesity (50.4% to 62.8%; p<0.001). By obesity subgroup, the prevalence increased 11.8 percentage points among persons with class 1 obesity (40.8% to 52.6%; p<0.001) and 15.5 percentage points among those with class 3 obesity (69.0% to 84.5%; p<0.001); the increase among persons with class 2 obesity was not significant (Figure). In 2014 among adults with arthritis and overweight or obesity, the prevalence of receiving provider weight-loss counseling was significantly higher for females (versus males), those with obesity (versus overweight), those who had ever received provider counseling to engage in physical activity to manage arthritis (versus those who had not), those who had ever taken a self-management class or course (versus those who had not), and those with a primary care provider (versus those without one) (Table).

**TABLE:** Age-standardized prevalence* of health care provider counseling for weight loss reported among adults aged ≥18 years with doctor-diagnosed arthritis and overweight or obesity, by selected characteristics — National Health Interview Survey, United States, 2002 and 2014

Characteristic	2002			2014			% change 2002 to 2014
	Unweighted no.	Weighted no. (x 1000) reporting counseling^†^	Age-standardized % (95% CI)	Unweighted no.	Weighted no. (x 1000) reporting counseling^†^	Age-standardized % (95% CI)	
**Overall**	**1,733**	**10,740**	**35.1 (33.0–37.3)**	**2,869**	**16,600**	**45.5 (42.9–48.1)**	**29.6^§^**
**Sociodemographic characteristics**							
**Age group (yrs) (age-specific)**							
18–44	246	1,599	30.9 (27.4–34.6)	399	2,570	47.1 (42.6–51.5)	52.4^§^
45–64	858	5,629	41.9 (39.4–44.4)	1,297	8,046	45.5 (42.8–48.2)	8.6
≥65	629	3,513	36.4 (34.0–38.9)	1,173	5,984	40.6 (38.2–43.1)	11.5^§^
**Sex**							
Male	592	4,444	31.3 (28.3–34.5)	1,028	6,670	41.1 (37.1–45.2)	31.3^§^
Female	1,141	6,297	38.6 (35.6–41.7)	1,841	9,930	49.2 (45.8–52.6)	27.5^§^
**Race/Ethnicity**							
Hispanic	1,168	8,061	32.9 (30.5–35.4)	1,887	12,033	44.0 (40.9–47.1)	33.7^§^
White, non-Hispanic	322	1,590	45.2 (39.2–51.3)	515	2,263	47.4 (41.8–53.1)	4.9
Black, non-Hispanic	209	825	38.5 (32.5–44.9)	364	1,865	54.0 (46.9–60.8)	40.3^§^
Other, non-Hispanic	34	265	44.0 (31.3–57.5)	103	439	42.0 (28.9–56.4)	−4.5
**Education**							
Less than HS graduate	423	2,183	31.3 (26.7–36.3)	527	2,567	41.7 (35.4–48.2)	33.2^§^
HS graduate or equivalent	535	3,461	34.3 (30.6–38.3)	776	4,728	45.9 (40.8–51.0)	33.8^§^
Technical school/Some college	458	2,905	35.2 (31.5–39.0)	913	5,417	47.1 (42.6–51.6)	33.8^§^
College degree or higher	306	2,128	37.9 (32.9–43.1)	645	3,818	44.1 (38.9–49.4)	16.4
**Work status**							
Employed	709	4,896	34.8 (32.0–37.8)	1,117	7,211	45.4 (42.1–48.7)	30.5^§^
Unemployed	33	191	25.5^¶^ (16.7–36.9)	111	697	45.8 (36.0–56.0)	79.6^§^
Unable to work/ Disabled	358	1,946	40.7 (35.5–46.1)	621	3,143	56.4 (50.2–62.4)	38.6^§^
Other	631	3,698	33.9 (27.2–41.3)	1019	5,546	39.6 (32.8–46.8)	16.8
**Health-related characteristic**							
**BMI (kg/m^2^)**							
Overweight (25 to <30)	482	3,023	18.1 (15.8–20.7)	743	4,352	23.8 (20.8–27.0)	31.5^§^
Obesity (≥30)	1,733	10,740	50.4 (47.3–53.6)	2,869	16,600	62.8 (59.6–65.9)	24.6^§^
Class 1 (≥30 to <35)	600	3,756	40.8 (36.7–45.0)	959	5,708	52.6 (48.0–57.2)	28.9^§^
Class 2 (≥35 to <40)	362	2,232	60.2 (54.7–65.4)	585	3,229	63.0 (56.3–69.2)	4.7
Class 3 (≥40)	289	1,729	69.0 (60.6–76.3)	582	3,311	84.5 (80.2–88.0)	22.5^§^
**Arthritis limitations**							
No	852	5,519	30.6 (28.1–33.2)	1,411	8,567	43.1 (39.8–46.4)	40.8^§^
Yes	878	5,206	42.5 (38.9–46.3)	1,457	8,029	48.7 (44.7–52.7)	14.6^§^
**Ever counseled by provider to engage in physical activity to manage arthritis**							
No	351	2,219	15.7 (13.5–18.2)	400	2,294	17.5 (14.5–21.0)	11.5
Yes	1,373	8,481	51.7 (48.5–54.9)	2,467	14,304	60.5 (57.1–63.7)	17.0^§^
Ever taken a self-management class or course**							
No	1,470	9,099	33.2 (31.0–35.5)	2,430	13,907	43.3 (40.6–46.1)	30.4^§^
Yes	262	1,639	50.7 (43.9–57.5)	439	2,693	61.5 (54.5–68.2)	21.3^§^
**Joint pain severity^††^**							
None or mild (0–4)	328	2,207	32.8 (28.5–37.5)	607	3,655	45.8 (39.7–51.9)	39.6^§^
Moderate (5–6)	406	2,688	35.5 (31.1–40.2)	669	3,967	49.2 (43.8–54.6)	38.6^§^
Severe (≥7)	615	3,396	42.9 (39.0–46.8)	960	5,389	47.8 (42.7–53.0)	11.4
**Self-rated health**							
Excellent/Very good	460	3,017	28.1 (25.1–31.4)	799	5,258	37.8 (33.7–42.0)	34.5^§^
Good	581	3,703	35.8 (31.9–39.9)	1,032	5,918	48.2 (43.6–52.8)	34.6^§^
Fair/Poor	692	4,021	45.7 (41.2–50.2)	1,037	5,419	55.1 (50.2–59.9)	20.6^§^
**Smoking status**							
Current smoker	273	1,716	30.4 (26.7–34.4)	444	2,413	39.7 (34.7–44.9)	30.6^§^
Former smoker	635	4,137	36.2 (31.7–41.0)	961	5,705	48.4 (42.3–54.5)	33.7^§^
Never smoker	823	4,868	37.0 (33.9–40.3)	1,461	8,474	46.8 (43.3–50.4)	26.5^§^
**Aerobic physical activity level^§§^**							
Active	509	3,490	33.9 (30.8–37.1)	941	5,715	42.2 (38.4–46.1)	24.5^§^
Insufficient	367	2,209	38.0 (32.9–43.4)	703	4,079	48.9 (43.1–54.9)	28.7^§^
Inactive	825	4,798	35.0 (31.7–38.5)	1,184	6,539	48.2 (43.5–52.8)	37.7^§^
**Have a primary care provider**							
No	133	709	30.8 (25.5–36.7)	190	947	32.1 (26.6–38.1)	4.2
Yes	1,600	10,032	36.0 (33.7–38.4)	2,678	15,649	47.6 (44.8–50.5)	32.2^§^
**No. of co-occurring chronic conditions^¶¶^**							
0	15	76	—***	49	311	51.4 (35.6–66.9)	—***
1–2	952	5,898	31.4 (29.1–33.8)	1,412	8,460	41.7 (38.7–44.7)	32.8^§^
≥3	766	4,767	49.4 (43.5–55.3)	1,408	7,829	52.8 (46.6–58.8)	6.9

**Abbreviations:** BMI = body mass index (kg/m^2^); CI = confidence interval; HS = high school.

* Estimates age-standardized to the 2000 U.S. standard population aged ≥18 years using three groups (18–44, 45–64, and ≥65 years).

^†^ Weighted number in thousands of adults with arthritis and overweight or obesity reporting counseling out of the total 28.3 million (2002) and 38.9 million (2014) adults with arthritis and overweight or obesity.

^§^ Difference is significant (p-value) at an α = 0.05 level.

^¶^ Estimate potentially unreliable: relative standard error between 20%–30%.

** Based on response to the question “Have you ever taken an educational course or class to teach you how to manage problems related to your arthritis or joint symptoms?”

^††^ Joint pain severity was categorized on a scale of 0 to 10 where 0 is no pain or aching and 10 is pain or aching as bad as it can be.

^§§^ Respondents were classified as active if they reported ≥150 minutes of moderate intensity leisure time aerobic physical activity per week, insufficiently active if they reported 1–149 minutes, and inactive if they reported 0 minutes. Reported vigorous intensity physical activity minutes were counted double and added to moderate intensity physical activity minutes.

^¶¶^ Among these nine chronic conditions: asthma, cancer, diabetes, heart disease, hepatitis, hypertension, kidney disease, serious psychological distress, and stroke.

*** Estimate is suppressed because of unstable relative standard error >30.0%.

**FIGURE:**
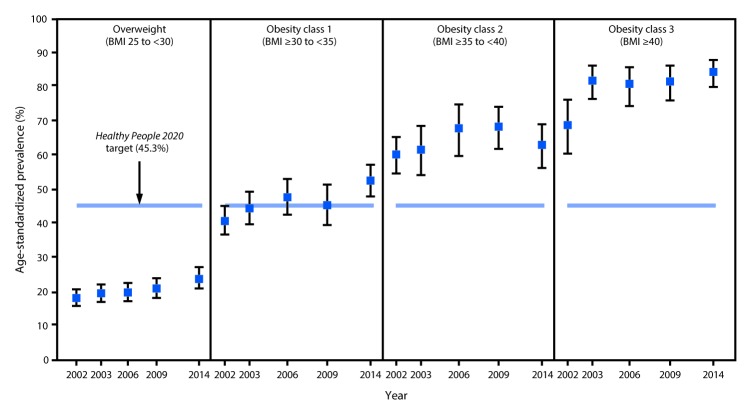
Age-standardized prevalence* of health care provider counseling for weight loss reported among adults aged ≥18 years with doctor-diagnosed arthritis and overweight or obesity, by year and body mass index (BMI) status — National Health Interview Survey, 2002, 2003, 2006, 2009, and 2014 * Estimates age-standardized to the 2000 U.S standard population aged ≥18 years using three age groups (18–44, 45–64, and ≥65 years).

## Discussion

From 2002 to 2014, the percentage of adults with arthritis and overweight or obesity who reported receiving provider weight-loss counseling increased by 10.4 percentage points. These improvements are encouraging; however, approximately 75% of adults with overweight and 50% of those with class 1 obesity are not receiving provider weight-loss counseling.

A recent report indicated that 61.0% of adults with arthritis received provider counseling for physical activity in 2014 (*7*), more than the 45.5% reported here for weight loss. Providers might advise for physical activity more frequently than weight loss because the former might be easier to discuss with patients or they might be more aware of the arthritis-specific benefits of physical activity. Findings of the current report indicate that those who are not receiving counseling for weight loss might also not be receiving counseling for physical activity. Nevertheless, to address obesity, the U.S. Preventive Services Task Force recommends that providers either provide or refer patients to intensive, multicomponent behavioral interventions that include management strategies (e.g., goal setting), dietary and physical activity changes, addressing barriers to change, self-monitoring, and strategies to maintain healthy behaviors.^§§^ The American College of Rheumatology also recommends that providers offer counseling for weight loss and physical activity to adults with hip or knee osteoarthritis. In randomized controlled trials, a combined exercise and diet intervention resulted in the greatest improvements in weight, pain, joint forces, inflammatory factors, and mobility compared with either intervention alone (*4*,*8*). In the current study, the percentage of adults with overweight or obesity who received weight-loss counseling was higher among those who had taken a self-management education course than among those who had not. Since the temporal sequencing of provider weight-loss counseling and taking a self-management education course (which includes weight-loss messages) cannot be delineated, this study could not determine whether provider counseling leads persons with arthritis and overweight or obesity to self-management education courses or vice versa. However, it is possible that persons with arthritis who receive recommendations for healthy behaviors, such as weight loss, from their provider are more amenable to engaging in other self-management behaviors, such as taking a self-management education course or engaging in physical activity.^¶¶^ One benefit of self-management education program participation is substantial increases in self-confidence (*9*), which is an important characteristic that can help adults with arthritis act on counseling to lose weight and be physically active. Combined counseling for weight loss, physical activity, and self-management education might enhance arthritis and other health outcomes.

Strategies to increase provider counseling for weight loss include health system interventions (e.g., electronic medical record clinical decision supports) and provider training. Electronic medical record clinical decision supports are effective in increasing the delivery of nutrition and physical activity counseling and decreasing BMI in children with obesity (*10*), and similar strategies might translate into weight loss in adult populations. Standardized electronic medical record clinical decision supports could assist provider counseling and referrals to evidence-based, community-delivered weight-loss and physical activity programs, intensive multicomponent interventions, or bariatric specialists, as well as facilitate patient education and help providers follow up on patients’ weight-loss goals and progress. Increased provider training regarding self-management support strategies can help providers to gain the skills and confidence to provide successful weight-loss counseling. Such training can include formal classroom instruction or use of publicly available online resources for counseling their patients.***^,^^†††^ Many effective strategies, including motivational interviewing, the 5As approach (Assess, Advise, Agree, Assist, and Arrange), and emphasizing that small changes can have a big impact, are applicable to weight-loss counseling (*6*). For example, along with improving pain and mobility (*4*), a relatively small, but clinically significant, 5.1% reduction in weight over 20 weeks can significantly reduce functional disability in patients with knee osteoarthritis and obesity (*5*).

The findings in this report are subject to at least four limitations. First, NHIS data are self-reported and some characteristics might be susceptible to recall or social desirability bias. Specifically, the latter can lead to underestimation of BMI (*2*). Second, low response rates could also introduce response bias; however, sampling weights applied in the analysis include adjustment for nonresponse. Third, using BMI to classify overweight and obesity risks classifying some persons with a high muscle-to-fat ratio as having overweight or obesity, who might not require counseling. Finally, because 2014 data for provider counseling for weight loss were the most recent available, the prevalence might have changed since then.

Reported receipt of provider counseling for weight loss increased significantly among adults with arthritis and overweight or obesity from 2002 to 2014. Continuing this progress can ensure that the majority of adults in this population receive important messages that can increase their attempts to lose weight. Through combined counseling for weight loss, physical activity, and self-management education, and by making referrals to evidence-based programs, providers can help their patients with arthritis make meaningful improvements in quality-of-life and long-term health outcomes.

**Summary**

**What is already known about this topic?**

Weight loss among adults with arthritis and overweight or obesity can improve pain, function, mobility, and health-related quality of life, and reduce disability.

**What is added by this report?**

From 2002 to 2014, the prevalence of health care provider counseling for weight loss among adults with arthritis and overweight or obesity increased by 10.4 percentage points from 35.1% to 45.5%.

**What are the implications for public health practice?**

Provider counseling for weight loss in adults with arthritis and overweight or obesity, along with other health behavior counseling, including physical activity and self-management education, might increase attempts at weight loss and eventual success.

## Footnotes

* https://onlinelibrary.wiley.com/doi/epdf/10.1002/acr.21596.

†
https://www.healthypeople.gov/2020/topics-objectives/topic/Arthritis-Osteoporosis-and-Chronic-Back-Conditions/objectives .

§
https://www.cdc.gov/nchs/nhis/data-questionnaires-documentation.htm .

¶
https://www.cdc.gov/obesity/adult/defining.html .

** https://www.nhlbi.nih.gov/files/docs/guidelines/prctgd_c.pdf.

†† https://www.cdc.gov/nchs/data/statnt/statnt20.pdf.

§§
https://www.uspreventiveservicestaskforce.org/Page/Name/tools-and-resources-for-better-preventive-care .

¶¶
https://downloads.cms.gov/files/cmmi/community-basedwellnessrrevention-sixthmnthoutcomes-operationalcostrpt.pdf .

***
http://stopobesityalliance.org/wp-content/themes/stopobesityalliance/pdfs/STOP-Provider-Discussion-Tool.pdf .

†††
https://health.mo.gov/living/healthcondiseases/obesity/pdf/Toolkit_Adult.pdf .
